# Survival Disparities in the Radiotherapeutic Management of Lung Cancer by Regional Poverty Level

**DOI:** 10.7759/cureus.3575

**Published:** 2018-11-12

**Authors:** Sean Mahase, Paul Christos, Xin Wang, Louis Potters, A. Gabriella Wernicke, Bhupesh Parashar

**Affiliations:** 1 Radiation Oncology, NewYork-Presbyterian/Weill Cornell Medical Center, New York, USA; 2 Biostatistics and Epidemiology, New York-Presbyterian/Weill Cornell Medical Center, New York, USA; 3 Radiation Oncology, Zucker School of Medicine at Hofstra / Northwell, New York, USA; 4 Radiation Medicine, Zucker School of Medicine at Hofstra / Northwell, New York, USA

**Keywords:** health disparities, radiation therapy, lung cancer, seer

## Abstract

Purpose

This study evaluates regional poverty level-dependent differences in lung cancer (LC) survival, focusing on patients receiving radiation therapy (RT).

Methods and materials

The Surveillance, Epidemiology, and End Results (SEER) database was used to retrospectively identify patients diagnosed with LC between 2000 and 2009. Patients were divided into socioeconomic status (SES) quintiles, with quintiles 1 and 5 representing the highest and lowest SES cohorts, respectively. The Kaplan-Meier method with the log-rank test was used to compare overall survival (OS) from diagnosis between demographic and clinical factor levels. Multivariate (MVA) Cox proportional hazards regression was used to examine the association of quintile and mortality, adjusting for demographic and clinical factors.

Results

Compared to those not receiving RT, the univariate (UVA) results showed a higher mortality associated with receiving RT (HR:1.091; CI:1.081-1.102) while the MVA demonstrated a protective effect (HR:0.882; CI:0.873-0.891). The MVA revealed that men had higher mortality rates than women (HR:1.192; CI:1.180-1.203). Caucasians had a lower mortality rate as compared to African Americans (adjusted HR:0.932; CI:0.918-0.947) while Asians, Pacific Islanders, and Native Americans had the highest overall survival rates (adjusted HR:0.752, CI:0.734-0.771). Among the entire study population, quintile 2 (HR:1.059, CI:1.043-1.076), quintile 3 (HR:1.091, CI:1.075-1.108), quintile 4 (HR:1.094, CI:1079-1.110), and quintile 5 (HR:1.201, CI:1.181-1.221) reported increased mortality rates compared with quintile 1. This trend was also observed among those undergoing RT, with quintile 2 (HR:1.034, CI:1.010-1.059), quintile 3 (HR:1.045; CI:1.021-1.069), quintile 4 (HR:1.056; CI:1.033-1.080), and quintile 5 (HR:1.153; CI:1.124-1.183) demonstrating incrementally worse OS.

Conclusions

Upon accounting for age, gender, race, SES, and tumor stage, RT may provide a positive survival benefit among those who received treatment. Minimal differences existed among SES quintiles regarding diagnoses made by tumor stage or patients receiving RT. An incrementally worse OS rate was associated with increasing regional poverty level. This trend persevered among those receiving RT.

## Introduction

Lung cancer (LC) is the third most common cancer diagnosis among men and women and the leading cause of cancer-related death in the United States. LCs were implicated in an estimated 159,260 reported deaths in 2014, accounting for 27.2% of all cancer-related deaths and surpassing the combined mortality of breast, colon, and prostate cancers. These sobering numbers are underscored by advances in surgical, chemotherapeutic, and radiation therapy (RT) yielding marginal improvements, with the five-year relative survival increasing from 11.4% in 1975 to 17.5% in 2006 [1].

While these execrable outcomes are principally due to LC’s virulent nature, there is an increasing interest in identifying modifiable co-morbidities affecting prognosis to improve survival outcomes. For instance, LC’s relatively asymptomatic presentation emphasizes the need for prompt diagnosis during its initial stages, which primarily relies on physical examination and imaging. Therefore, any disparities affecting health care accessibility considerably influences one’s ability to be diagnosed and receive subsequent treatment, drastically affecting their clinical outcome.

Numerous empirical investigations explored and documented disparities in LC incidence and diagnosis [2], access to treatment [3], and survival [4-5]. Most focus on potential disparities among ethnicities, insurance and Medicaid status, geography, socioeconomic status (SES), and education level. Shugarman and colleagues reported no correlation between residing in a rural environment and survival [6]. A systematic review by the American Thoracic Society revealed patients with Medicaid or no insurance had higher LC incidence rates and were diagnosed at later tumor stages, correlating with poorer overall survival (OS) rates compared to those with private insurance or Medicare [7]. Other investigations demonstrate lower SES is associated with poorer OS in LC in other countries [8] as well as in the United States [9]. The aforementioned and other studies yielded important results with respect to the impact of these potential disparities on the multidisciplinary approach of LC in America. However, there is a dearth of studies examining social inequalities exclusively among patients who underwent treatment following diagnosis. Ascertaining the existence of these potential disparities becomes increasingly important as more Americans, particularly those of lower SES, gain treatment access through the Affordable Care Act.

In this study, we analyze the relationship of regional poverty level with race, age, gender, and tumor stage to determine whether SES-dependent disparities exist. We then analyze the independent effect of SES disparities on the OS of LC patients, as well as on those who received RT, after adjusting for influential confounders. To our knowledge, this is the first report demonstrating that regional poverty level-dependent disparities in OS exist across the United States among LC patients, and persevere specifically among those who undergo RT.

## Materials and methods

Participants, data selection, and study design

The National Cancer Institute 18 Surveillance, Epidemiology, and End Results (SEER) program state and regional population-based cancer registries were used to obtain information regarding 386,551 lung and bronchus cancer diagnoses (International Classification of Diseases for Oncology, 3rd Edition histology codes 8000-8005, 8010-8015, 8020-8022, 8030-8035, 8041-8046, 8050-8052, 8070-8076, 8078, 8120-8124, 8140, 8141, 8143, 8147, 8200, 8201, 8230, 8231, 8240-8246, 8249-8255, 8260, 8310, 8320, 8323, 8430, 8480, 8481, 8490, 8510, 8550, 8551, 8560, 8562, 8570-8576, 8800-8806, 8810, 8811, and 8813-8815) [10]. SEER collects demographic, tumor-related, treatment-related, and follow-up information from 18 regions in the United States, accounting for approximately 27.8% of the United States population as per the 2010 census.

SES was measured as the percentage of persons living below the federal poverty line in a patient’s county of residence. All 3,143 counties in the SEER database were sequentially listed by reported percentages of persons below poverty residing in each respective county in 2000. These counties were divided into roughly equal quintiles, such that quintiles 1, 2, 3, 4, and 5 represented counties reporting 0-7.63%, >7.63-10.33%, >10.33-14.13%, >14.13-17.91%, and >17.91%-45.38% of their respective populations living under the federal poverty line. Every patient was linked to their county of residence and placed into one of the five poverty groupings.

Individual-level variables of interest included SES quintile, gender, race, age and tumor stage at diagnosis, and type of RT received. To reflect the impact of recent and rapid advancements in LC-directed RT modalities [11] while maintaining a population with adequate statistical power, patients diagnosed with LC, regardless of RT status, from January 2000 to December 2009 were chosen. Patient data were age-standardized to the International Cancer Survival Standard derived by Corazziari et al. [12]. Staging at diagnosis was based on the American Joint Committee on Cancer (AJCC), 6th edition [13]. RT was defined as receiving external beam radiation (EBRT), radioactive implants, radioactive isotopes, combination EBRT with implants and isotopes, and RT not otherwise specified (NOS). Patients not receiving RT, and who refused treatment, were tallied for each SES group. Patients with unknown tumor stage or unknown county of residence were excluded, providing a final study population of 200,962.

Statistical analysis

Descriptive statistics for factors of interest were reported as n (%) for categorical measures and as mean, median and standard deviation for continuous measures. Factors were compared between the poverty quintiles using chi-square tests and analysis of variance (ANOVA) as applicable. Chi-square tests, two-sample t-tests, and ANOVA were employed to analyze bivariate relationships of interest. The main outcome was death due to any cause, and time to death was defined as time from date of diagnosis to either the date of death or the date of last follow-up/data collection. Patients were censored at the date of last follow-up/data collection if they had no record of death in the SEER database. Kaplan-Meier survival analyses employing the log-rank test were performed to evaluate OS differences by demographic/clinical factor levels of interest. Unadjusted (UVA) Cox proportional hazards regressions were performed to evaluate the hazard of death due to any cause as a function of the poverty quintile and of other factors of interest. Multivariate (MVA) Cox proportional hazards regression was constructed with all factors to evaluate the independent effects of poverty level and receipt of RT on OS. Specifically, two MVAs were constructed in order to show RT results with two separate reference categories. Subsequently, UVA and MVA Cox proportional hazards regressions were performed just within the cohort of patients who received RT. All tests were two-sided and considered significant at the 0.05 alpha level. All analyses were performed in R (3.2.1) for Windows.

## Results

Patient demographics and clinical variables

Between 2000 and 2009, 200,962 patients were diagnosed with LC (Table 1). Overall, our cohort entailed a higher proportion of males (53.8%) than females. However, the proportion of male patients increased with the poverty level: quintile 1 had a 2% greater male population while quintile 5 had a 16% greater male population. The majority of patients were Caucasian (82.6%), with African American and ‘other’ (Native Americans, Asians, and Pacific Islanders) comprising 11.3% and 6.0% of the study population, respectively. The proportion of Caucasian and ‘other’ patients in each quintile generally trended down with poverty level while the proportion of African American patients per quintile increased with the poverty level. On average, women were diagnosed one year older (mean age 68.64) than men (Table 2). The age of diagnosis trended down with increasing poverty level, with quintiles 1 and 5 reporting means of 68.60 and 66.95 years old, respectively. There were minor but significant differences among SES quintiles in the proportions of diagnoses made at each AJCC stage.

**Table 1:** Patient Demographics and Clinical Variables by SES Quintile Patient demographics and clinical variable by SES quintile. * Percentage of persons living below federal poverty line within each patient’s county of residence. SES: socioeconomic status; AJCC: American Joint Committee on Cancer; n: number of patients

Categorical Variable		Quintile 1 (0.00-7.63%)*	Quintile 2 (>7.63 - 10.33)	Quintile 3 (>10.33-14.13)	Quintile 4 (>14.13-17.91)	Quintile 5 (>17.91-45.38)	p-value
Gender	Female	20790 (49%)	17979 (47%)	19250 (46%)	23401 (45%)	11378 (42%)	<0.001
	Male	21295 (51%)	20497 (53%)	22276 (54%)	28104 (55%)	15992 (58%)	
AJCC Stage	0	25 (0%)	32 (0%)	20 (0%)	35 (0%)	18 (0%)	
	IA and IB	8103 (19%)	7180 (19%)	7490 (18%)	8724 (17%)	4618 (17%)	
	IIA and IIB	1978 (5%)	1715 (4%)	1856 (4%)	2176 (4%)	1287 (5%)	<0.001
	IIIA and IIIB	10451 (25%)	10088 (26%)	11027 (27%)	13355 (26%)	7798 (28%)	
	IV	21005 (50%)	19014 (49%)	20629 (50%)	26481 (51%)	13052 (48%)	
	OCCULT	523 (1%)	447 (1%)	504 (1%)	734 (1%)	597 (2%)	
Radiation	External beam radiation	16369 (39%)	14954 (39%)	16011 (39%)	18802 (37%)	10630 (39%)	
	Combination of beam with implants or isotopes	66 (<1%)	33 (<1%)	46 (<1%)	72 (<1%)	41 (<1%)	
	None	24872 (59%)	22562 (59%)	24226 (58%)	31249 (61%)	15767 (58%)	<0.001
	Radiation, method or source not specified	222 (1%)	295 (1%)	346 (1%)	366 (1%)	368 (1%)	
	Radioactive implants	71 (<1%)	41 (<1%)	41 (<1%)	62 (<1%)	38 (<1%)	
	Radioisotopes	7 (<1%)	9 (<1%)	11 (<1%)	7 (<1%)	5 (<1%)	
	Refused	478 (1%)	582 (2%)	845 (2%)	947 (2%)	521 (2%)	
Radiation	Refused	478 (1%)	582 (2%)	845 (2%)	947 (2%)	521 (2%)	<0.001
	None	24872 (59%)	22562 (59%)	24226 (58%)	31249 (61%)	15767 (58%)	
	Radiation	16735 (40%)	15332 (40%)	16455 (40%)	19309 (37%)	11082 (40%)	
Race	African American	2240 (5%)	2257 (6%)	4180 (10%)	9575 (19%)	4531 (17%)	<0.001
	Other (American Indian/Alaskan Native, Asian/Pacific Islander)	1943 (5%)	3733 (10%)	3041 (7%)	3039 (6%)	396 (1%)	
	Caucasian	37902 (90%)	32486 (84%)	34305 (83%)	38891 (76%)	22443 (82%)	

**Table 2:** Age at Diagnosis *Percentage of persons living below the federal poverty line within the patient’s county of residence

Categorical Variable		Number of Patients	Mean Age	Standard Deviation	Median Age	p-value
	1 (0.00-7.63%)*	42085	68.60	11.39	69	
	2 (>7.63 - 10.33)	38476	68.44	11.41	69	
Quintile	3 (>10.33-14.13)	41526	68.23	11.33	69	<0.001
	4 (>14.13-17.91)	51505	67.96	11.29	69	
	5 (>17.91-45.38)	27370	66.95	10.97	67	
Gender	Female	92,798	68.64	11.61	69	<0.001
	Male	108,164	67.64	11.02	68	

Radiation therapy

Of all patients diagnosed with LC, 59.1% (n=118,676) did not receive RT, 39.3% (n=78,913) underwent RT, and 1.6% (n=3373) refused RT despite being recommended for treatment. The patients who did not receive RT (mean = 69.54 years old), and who refused to undergo RT (mean = 73.34 years old), were older than all groups who underwent various RT modalities (Table 3). EBRT was used in 97.3% of all patients who underwent RT, with under 1% of patients receiving radioisotopes, radioactive implants or combination EBRT, and implants or isotopes. Another 2% of patients received RT whose modality was not specified in the SEER database (Table 4). Analyzing the AJCC stage at diagnosis (Table 5), late-stage (III and IV) LC trended towards a higher likelihood of receiving RT than early stage (I and II) LC. Stage IIIA had a greater proportion of patients undergoing RT than stages IIIB and IV. Additionally, a larger proportion of patients who refused RT were diagnosed at later stages. Analyzing RT by SES for stage IV patients (Table 6) revealed minor but significant differences among quintiles with respect to the proportion of patients receiving RT and the number of patients refusing RT.

**Table 3:** Radiation Treatment by Age at Diagnosis

Radiation	Number of Patients	Mean Age	Standard Deviation	Median Age	p-value
External beam radiation	76766	65.73	11.06	66	<0.001
Combination of beam with implants or isotopes	258	63.39	10.7	63	
Radiation, method, or source not specified	1597	65.59	10.96	66	
Radioactive implants	253	67.43	10.36	68	
Radioisotopes	39	67	11.17	70	
Refused	3373	73.34	10.93	75	
None	118676	69.54	11.2	70	

**Table 4:** Radiation Modalities Amongst Those Who Received Radiation

Radiation Modality	Number of Patients (%)
External beam radiation	76,766 (97.28%)
Combination of beam with implants or isotopes	258 (0.33%)
Radiation, NOS method or source not specified	1,597 (2.02%)
Radioactive implants	253 (0.32%)
Radioisotopes	39 (0.05%)
Total	78,913

**Table 5:** Radiation Treatment by AJCC Stage AJCC: American Joint Committee on Cancer 6^th^ edition

AJCC Stage Group	Received Radiation (all modalities)	Beam Radiation	Radioactive implants	Radioisotopes	Combination beam with implants or isotopes	Radiation, method, or source not specified	None	Refused
0	25 (19%)	20 (15%)	5 (4%)	0 (<1%)	0 (<1%)	0 (<1%)	103 (79%)	2 (2%)
IA	2,899 (16%)	2,800 (15%)	37 (<1%)	3 (<1%)	6 (<1%)	53 (<1%)	15,492 (83%)	181 (<1%)
IB	3,754 (21%)	3,622 (21%)	30 (<1%)	1 (<1%)	18 (<1%)	83 (<1%)	13,548 (77%)	241 (1%)
IIA	563 (28%)	548 (27%)	2 (<1%)	0 (<1%)	1 (<1%)	12 (<1%)	1,444 (71%)	25 (1.%)
IIB	2,737 (39%)	2,658 (38%)	5 (<1%)	1 (<1%)	14(<1%)	59 (<1%)	4,160 (60%)	83 (1%)
IIIA	11,005 (58%)	10,765 (56%)	29 (<1%)	4 (<1%)	35 (<1%)	172 (<1%)	7,721 (40%)	354 (2%)
IIIB	15,316 (46%)	14933 (44%)	52 (<1%)	4 (<1%)	67 (<1%)	260 (<1%)	17,669 (52%)	654 (2%)
IV	41,961 (42%)	40,806 (41%)	88 (<1%)	25 (<1%)	113 (<1%)	929 (<1%)	56,454 (56%)	1,766 (2%)
OCCULT	653 (23%)	614 (22%)	5 (<1%)	1 (<1%)	4 (<1%)	29 (1%)	2,085 (74%)	67 (2%)
Total (all stages)	78,913	76,766	253	39	258	1,597	118,676	3,373

**Table 6:** Radiation Treatment by SES Quintiles for AJCC Stage IV Patients SES: socioeconomic status; AJCC: American Joint Committee on Cancer 6^th^ edition; n: number of patients

Quintile	1	2	3	4	5	p-value
Refused Radiation	241 (1%)	304 (2%)	430 (2%)	518 (2%)	273 (2%)	<0.001
None	11613 (55%)	10476 (55%)	11570 (56%)	15509 (59%)	7286 (56%)	
Received Radiation	9151 (44%)	8234 (43%)	8629 (42%)	10454 (39%)	5493 (42%)	

Survival

**Figure 1:**
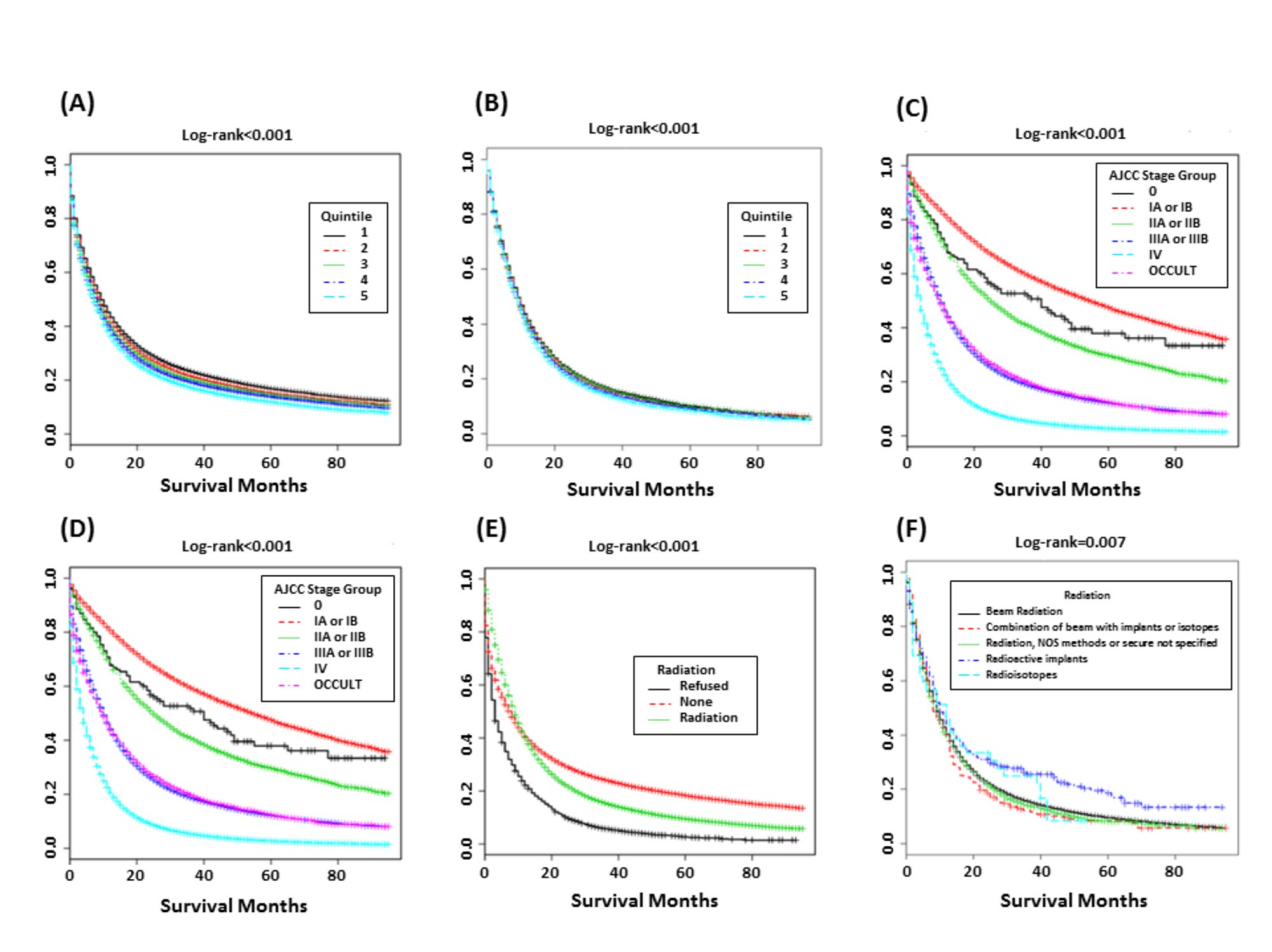
Overall Survival Kaplan-Meier estimates of overall survival for: (A) All patients by poverty quintile, (B) Patients receiving radiation by quintile, (C) All patients by AJCC stage group, (D) Patients receiving radiation by AJCC stage group, (E) Treatment modality, and (F) Type of radiation received AJCC: American Joint Committee on Cancer

**Figure 2:**
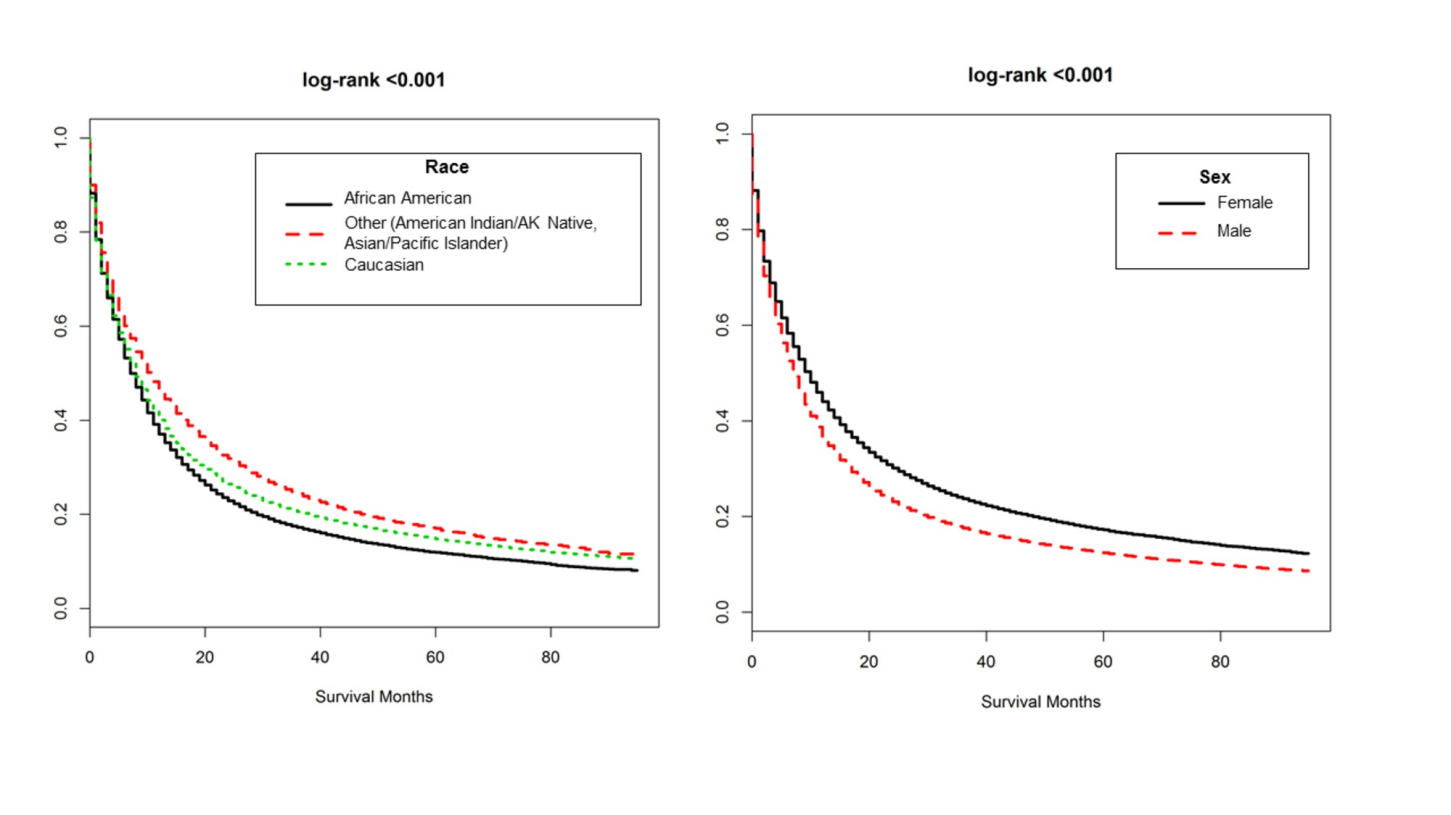
Overall Survival by Race and Sex Kaplan-Meier estimates of overall survival for all patients by: (A) Race and (B) Sex.

Kaplan-Meier curves of OS by poverty quintile, AJCC stage, and treatment modality for all patients, and for those receiving RT, are respectively shown in Figures 1A-1F. Patients in quintiles 1 and 5 trended towards the lowest and highest mortality rates, respectively (log-rank <0.001). This trend persevered among those receiving RT (Table 7), with five-year OS incrementally decreasing with worsening SES. Caucasians demonstrated lower mortality rates than African Americans but higher than that of ‘other’ ethnicities (Figure 2A). Males had higher mortality rates than females (Figure 2B). Mortality rate generally increased with tumor stage. Overall, stage 0 patients demonstrated higher mortality rates to patients with stage I disease; however those in stage 0 receiving RT demonstrated lower mortality rates than those receiving RT in later tumor stages. Patients who refused treatment had the highest mortality rates while the lowest mortality rates were seen among those not receiving RT.

**Table 7:** Five-year Overall Survival for Lung Cancer Patients Receiving Radiation Therapy 5-year overall survival for lung cancer patients receiving radiation therapy. *Percentage of persons living below the federal poverty line within a patient’s county of residence n: number of patients

Cohort	Overall survival (%) (95% CI)
All Quintiles (n=78,913)	9.5% (9.3%-9.8%)
Individual Quintile*	
1	10.0% (9.6%-10.6%)
2	9.7% (9.2%-10.2%)
3	9.9% (9.4%-10.4%)
4	9.2% (8.8%-9.7%)
5	8.5% (7.9%-9.1%)

Regression analysis

Tables 8-9 present the UVA and MVA Cox proportional hazards regression results for the entire population and those receiving RT, respectively. According to the UVA for the entire population, the hazard of death increased with increasing poverty level, with those in quintiles 2 and 5, respectively, having a 5% (HR:1.048; CI:1.032-1.064) and 20% (HR:1.196; CI:1.176-1.216) higher hazards of death than patients in quintile 1. Males had a higher mortality rate than females (HR:1.190; CI:1.179-1.202). Caucasians and patients grouped as ‘other’ ethnicities had lower mortality rates compared to African Americans (HR: 0.921; CI: 0.908-0.935 and HR: 0.817; CI: 0.797-0.837, respectively). A later tumor stage at diagnosis correlated with higher mortality rates than those at stage 0, with stage II (HR: 1.237; CI:0.986-1.551), stage III (HR: 2.268; CI:1.811-2.841), and stage IV (HR: 4.159; CI:3.321-5.208), demonstrating incrementally higher hazards of death compared to those in stage 0. For each year increase in age at diagnosis, the hazard of death increased on average by about 2% (HR: 1.015; CI: 1.014, 1.105). Compared to not receiving RT, the UVA showed a higher hazard of death associated with receiving ‘any RT’ (HR:1.091; CI:1.081-1.102) or refusing treatment (HR:1.785; CI:1.724-1.849). These trends in sex, tumor stage, and race persevered among those receiving RT, with those in quintiles 2 (HR:1.016; CI:0.993-1.040) and 5 (HR:1.092; CI:1.065-1.121) undergoing RT, respectively, having higher hazards of death than those undergoing RT in quintile 1. Compared to undergoing EBRT, the UVA showed a lower hazard of death associated with radioactive implant treatment (HR:0.799; CI:0.696-0.918). There were no significant differences in survival seen among the other RT modalities.

**Table 8:** Cox Proportional Hazards Regression Results for All Patients n=200,962; *Same univariate and multivariable analyses but with ‘refused’ used as reference group for radiation; HR: hazard ratio; CI: confidence interval; AJCC: American Joint Committee on Cancer 6^th^ edition

	Univariate Analyses				Multivariable Analyses				
Categorical Variable	HR	lower CI	upper CI	p-value	HR	lower CI	upper CI	p-value	Reference Group
Quintile									Quintile 1
2	1.048	1.032	1.064	<0.001	1.059	1.043	1.076	<0.001	
3	1.09	1.073	1.106	<0.001	1.091	1.075	1.108	<0.001	
4	1.127	1.111	1.143	<0.001	1.094	1.079	1.110	<0.001	
5	1.196	1.176	1.216	<0.001	1.201	1.181	1.221	<0.001	
Sex									Female
Male	1.19	1.179	1.202	<0.001	1.192	1.180	1.203	<0.001	
AJCC Stage									AJCC Stage 0
IA and IB	0.762	0.608	0.955	0.018	0.761	0.608	0.954	0.018	
IIA and IIB	1.237	0.986	1.551	0.066	1.312	1.047	1.645	0.019	
IIIA and IIIB	2.268	1.811	2.841	<0.001	2.475	1.976	3.100	<0.001	
IV	4.159	3.321	5.208	<0.001	4.618	3.688	5.783	<0.001	
OCCULT	2.289	1.821	2.876	<0.001	2.203	1.753	2.768	<0.001	
Radiation									Refused Radiation Treatment
None	0.56	0.541	0.58	<0.001					
Beam radiation	0.611	0.59	0.633	<0.001					
Combination of beam with implants or isotopes	0.65	0.57	0.743	<0.001					
Radiation, method, or source not specified	0.629	0.591	0.669	<0.001					
Radioactive implants	0.503	0.437	0.58	<0.001					
Radioisotopes	0.583	0.416	0.817	0.002					
Radiation									None
Refused	1.785	1.724	1.849	<0.001	1.415	1.366	1.466	<0.001	
Radiation (Any Modality)	1.091	1.081	1.102	<0.001	0.882	0.873	0.891	<0.001	
Race									African American
Other	0.817	0.797	0.837	<0.001	0.752	0.734	0.771	<0.001	
Caucasian	0.921	0.908	0.935	<0.001	0.932	0.918	0.947	<0.001	
Age at diagnosis	1.015	1.014	1.105	<0.001	1.021	1.0203	1.0212		

**Table 9:** Cox Proportional Hazards Regression Results for Patients Receiving Radiation Therapy n=78,913; HR: hazard ratio; CI: confidence interval; AJCC: American Joint Committee on Cancer 6th edition

	Univariate Analyses				Multivariable Analyses				
Categorical Variable	HR	lower CI	upper CI	p-value	HR	lower CI	upper CI	p-value	Reference Group
Quintile									Quintile 1
2	1.016	0.993	1.040	0.173	1.034	1.010	1.059	0.005	
3	1.012	0.989	1.036	0.291	1.045	1.021	1.069	<0.001	
4	1.048	1.025	1.072	<0.001	1.056	1.033	1.080	<0.001	
5	1.092	1.065	1.121	<0.001	1.153	1.124	1.183	<0.001	
Sex									Female
Male	1.171	1.154	1.189	<0.001	1.174	1.157	1.192	<0.001	
AJCC Stage									AJCC Stage 0
IA and IB	1.473	0.872	2.488	0.148	1.504	0.889	2.546	0.128	
IIA and IIB	1.626	0.962	2.750	0.070	1.799	1.063	3.047	0.029	
IIIA and IIIB	2.143	1.269	3.619	0.004	2.426	1.435	4.103	0.001	
IV	4.729	2.800	7.985	<0.001	5.573	3.295	9.425	<0.001	
OCCULT	2.058	1.211	3.498	0.008	2.150	1.263	3.659	0.005	
Radiation									Beam Radiation
Combination of beam with implants or isotopes	1.081	0.951	1.229	0.231	1.194	1.050	1.357	0.007	
Radiation, method or source not specified	1.034	0.981	1.090	0.210	0.975	0.925	1.027	0.337	
Radioactive implants	0.799	0.696	0.918	0.002	1.003	0.873	1.152	0.968	
Radioisotopes	0.939	0.671	1.315	0.715	0.902	0.644	1.263	0.548	
Race									African American
Other	0.881	0.848	0.915	<0.001	0.787	0.757	0.818	<0.001	
Caucasian	0.981	0.959	1.003	0.091	0.959	0.937	0.981	<0.001	
Age at diagnosis	1.007	1.007	1.008	<0.001	1.016	1.015	1.017	<0.001	

According to the MVA, when adjusting for AJCC staging, sex, quintile, race, and age, the hazard ratio for receiving ‘any RT’ changed from that seen in the UVA, suggesting a protective effect (HR:0.882; CI:0.873-0.891). Refusing RT continued to be associated with increased mortality (HR:1.415; CI:1.366-1.466) compared to those receiving RT. The hazard ratios for quintile 2 (HR:1.059, CI=1.043, 1.076), quintile 3 ( HR:1.091; CI:1.075-1.108), quintile 4 (HR:1.094; CI:1079-1.110), and quintile 5 (HR:1.201; CI:1.181-1.221) collectively demonstrated incrementally worse OS associated with increasing poverty level. Additionally, being diagnosed at later stages correlated with higher mortality rates. Males continued to have higher mortality rates than females (HR:1.192; CI:1.180-1.203), while ‘other’ (HR: 0.752; CI: 0.734-0.771) and Caucasians (HR: 0.932; CI:0.918-0.947) continued to have lower mortality rates than African Americans. For each year increase in age at diagnosis, the hazard of death increased on average by 2% (HR: 1.021; CI: 1.0203, 1.0212). These trends in tumor stage, sex, and race persevered among those who received RT, with those undergoing RT in quintile 2 (HR:1.034, CI:1.010-1.059), quintile 3 (HR:1.045; CI:1.021-1.069), quintile 4 (HR:1.056; CI:1.033-1.080), and quintile 5 (HR:1.153; CI:1.124-1.183) demonstrating incrementally worse OS associated with increasing poverty level. Compared with EBRT, combination RT (HR:1.194; CI:1.050-1.357) and radioisotopes (HR:0.902; CI:0.644-1.263) continued to be associated with higher mortality and lower mortality, respectively, while radioactive implants (HR:1.003; CI:0.873-1.152) approached a similar hazard of death and RT NOS (HR:0.975; CI:0.925-1.027) demonstrated a lower hazard of death.

## Discussion

This report provides a descriptive analysis of the impact of disparities on LC survival, with attention to those receiving RT. Consistent with reports from several studies [9,14-15], the majority of LC diagnoses continue to be made notoriously later in the disease pathogenesis, with 50% of all initial diagnoses made at stage IV, and 76% of all cases diagnosed at stage III or IV. There were minor but significant differences among SES quintiles in the proportions of diagnoses made at each AJCC stage. The proportion of Caucasian and ‘other’ (Native Americans, Asians, Pacific Islanders) patients in each respective quintile generally trended down with poverty level while the proportion of African American patients per quintile increased with poverty level. While our overall study population consisted of a greater proportion of males, the relative proportion of each quintile’s male constituency increased with poverty level. This brings into question whether women are being appropriately selected for LC screening in regions of lower SES.

The patients who did not receive RT and who refused to undergo RT were older than all groups who underwent various RT modalities. Additionally, late stage LC cases trended towards a higher likelihood of receiving RT than early stage LC cases. Collectively, these observations likely reflect clinical judgments weighing the benefits of undergoing RT in the context of the respective patient’s expected long-term prognosis. Minimal differences existed among SES quintiles with respect to the proportions of patients diagnosed at stage IV who were offered RT and who refused RT.

Women had superior survival to men, consistent with prior reports that women had better survival than men regardless of the type of treatment received, possibly attributed to gender differences in tumor development and biology [16]. In agreement with findings by Tannenbaum and colleagues [9], Asians and Pacific Islanders demonstrated the lowest mortality rates, followed respectively by Caucasians and African Americans. Compared to not receiving RT, the UVA showed a higher hazard of death associated with receiving ‘any RT.’ However, when adjusting for other clinical parameters in the MVA, the hazard ratio for receiving ‘any RT’ flips, suggesting a protective effect on survival. This observation may be secondary to the aforementioned role of clinical judgment when deciding to offer a patient RT, accounting for patients at later stages having improved survival with RT while healthier patients, or those who would likely not benefit from RT, are not recommended for treatment.

LC patients who underwent RT in affluent regions demonstrated incrementally improved OS rates compared with their cohorts living in impoverished regions, consistent with prior studies showing living in areas of higher deprivation is associated with poor LC survival [6,9,14,17-18]. In particular, the highest mortality rates were observed among quintiles 4 and 5. These findings parallel a retrospective cohort study of LC cases in a Delaware tumor registry that reported no significant disparities existed between patients in the three higher SES groups but associated lower survival with being in the lowest SES group [15]. Rengen et al. reported greater utilization of emerging RT approaches in patients living in regions with lower poverty rates and lower unemployment rates among LC patients treated at 45 RT facilities between 2006 and 2007 [19]. This warrants evaluating whether patients in impoverished regions have access to newer RT techniques and modalities that may improve treatment outcomes. The United States government is actively working towards recognizing and remediating the underlying causes of disparities in LC treatment [20-21]. In light of these endeavors, disparities in health care access, screening, and treatment availability, as well as behavioral and occupational factors are growing public health concerns impacting diagnosis, treatment, and follow-up.

Patients were placed into quintiles based on their county of residence’s poverty rate, which may not accurately portray individual financial status, healthcare management, and subsequent survival. This information is difficult to obtain considering SEER documents whether a patient received RT but does not provide the time between diagnosis and RT, confirm undergoing the entire treatment course, treatment tolerance, or survival following completing RT. While several individual-level demographic variables were accounted for, insurance status, marital status, education level, ability to travel to health care facilities, and the management of other co-morbidities (e.g. smoking status, diabetes, cardiovascular disease) can impact survival and are potential sources of social inequality to be considered in future analyses building upon these findings.

## Conclusions

We demonstrate that upon accounting for age, gender, race, SES, and tumor stage, RT may provide a positive survival benefit among LC patients who received treatment. However, while minimal differences exist among counties of differing poverty rates regarding diagnoses made by tumor stage, or patients receiving RT, regional poverty level-dependent disparities exist with respect to OS, including among those undergoing RT. Furthermore, these SES disparities in OS persevere after accounting for other demographic and clinical factors. Underlying reasons for these disparities in lower SES regions may include lower follow-up rates (and inadequate medical management of treatment-related adverse effects), unidentified and unmanaged medical comorbidities, or differences in quality of living and social support. Clinicians and public health officials aware of the importance of regional SES factors should strive to improve LC treatment outcomes in impoverished areas. Additionally, these results warrant investigating whether SES-dependent disparities in OS exist among those undergoing other treatment modalities (e.g. surgery) in LC and those receiving RT for other malignancies.
